# The evolution of selective autophagy as a mechanism of oxidative stress response: The evolutionarily acquired ability of selective autophagy receptors to respond to oxidative stress is beneficial for human longevity

**DOI:** 10.1002/bies.202300076

**Published:** 2023-08-21

**Authors:** Joshua Ratliffe, Tetsushi Kataura, Elsje G. Otten, Viktor I. Korolchuk

**Affiliations:** ^1^ Biosciences Institute, Faculty of Medical Sciences Newcastle University Newcastle upon Tyne UK; ^2^ Present address: Amphista Therapeutics Cambridge UK

**Keywords:** ageing, aggrephagy, autophagy, mitophagy, neurodegeneration, oxidative stress, selective autophagy receptors

## Abstract

Ageing is associated with a decline in autophagy and elevated reactive oxygen species (ROS), which can breach the capacity of antioxidant systems. Resulting oxidative stress can cause further cellular damage, including DNA breaks and protein misfolding. This poses a challenge for longevous organisms, including humans. In this review, we hypothesise that in the course of human evolution selective autophagy receptors (SARs) acquired the ability to sense and respond to localised oxidative stress. We posit that in the vicinity of protein aggregates and dysfunctional mitochondria oxidation of key cysteine residues in SARs induces their oligomerisation which initiates autophagy. The degradation of damaged cellular components thus could reduce ROS production and restore redox homeostasis. This evolutionarily acquired function of SARs may represent one of the biological adaptations that contributed to longer lifespan. Inversely, loss of this mechanism can lead to age‐related diseases associated with impaired autophagy and oxidative stress.

## Graphical abstract

Ageing is associated with mitochondrial dysfunction, elevated reactive oxygen species (ROS) and loss of protein homeostasis. Longevous organisms, such as humans, evolved oxidation‐sensitive selective autophagy receptors (SARs), which are activated by forming disulphide‐linked conjugates (DLC) in the vicinity of dysfunctional mitochondria and protein aggregates. Resulting selective autophagic degradation of the sources of oxidative stress acts as an antioxidant system.

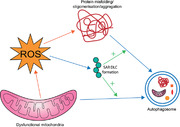

**Abbreviations**
AD: Alzheimer's disease
ALS: amyotrophic lateral sclerosis
CC: coiled coil
DLC: disulphide‐linked conjugate
KIR: KEAP1‐binding region
LIR: LC3 interacting region
MICOS: mitochondrial contact site and cristae organising system
NOX: NADPH oxidase
OPTN: optineurin
PD: Parkinson's disease
POAG: primary open angle glaucoma
ROS: reactive oxygen species
SAM: sorting and assembly machinery
SAR: selective autophagy receptor
UBD: ubiquitin binding domain

## INTRODUCTION

Autophagy is the process by which cells remove unnecessary or dysfunctional components through a lysosome‐dependent pathway. Autophagy was initially characterised as a non‐selective starvation‐induced bulk degradation response,^[^ ^1^, ^2^, ^3^ ^]^ however over the years it has been established that in order to maintain cellular homeostasis autophagy can selectively degrade specific cargo, such as damaged organelles, misfolded aggregate‐prone proteins and pathogens.^[^ ^4^ ^]^ There are three main forms of autophagy: macroautophagy, microautophagy and chaperone‐mediated autophagy. The macroautophagy pathway sequesters cytosolic components into a double‐membrane vesicle known as the autophagosome, which then fuses with the lysosome to degrade the cargo. As the most studied autophagy pathway, macroautophagy will hereafter be referred to simply as autophagy.^[^ ^4^ ^]^

Autophagy deficiency is a well‐established feature of ageing, with autophagic activity decreasing with age,^[^ ^5^ ^]^ and autophagy dysfunction playing an important role in many of the characteristic changes seen in ageing such as loss of proteostasis, genome instability and telomere exhaustion, among others.^[^ ^6^ ^]^ Evidence from animal models including *C. elegans*,^[^ ^7^, ^8^, ^9^ ^]^ *D. melanogaster* ^[^ ^10^, ^11^, ^12^ ^]^, and across different mouse tissues including brain and muscle^[^ ^13^, ^14^, ^15^, ^16^ ^]^ has shown that autophagy inhibition shortens lifespan across various organisms, whilst enhancing autophagy can extend lifespan.^[^ ^17^ ^]^

Ageing is also commonly associated with oxidative stress, which results from the accumulation of reactive oxygen species (ROS). Although a physiological level of ROS is necessary for redox signalling, with specific ROS molecules such as hydrogen peroxide (H_2_O_2_) and the superoxide anion radical (O_2_^.−^) acting as signalling agents through specific protein targets,^[^ ^18^ ^]^ excessive ROS levels can cause widespread molecular damage inside cells, including DNA mutations and damage to proteins and lipids. These changes resulting from elevated ROS may accumulate over time and can contribute to the classical hallmarks of ageing such as genome instability and the loss of proteostasis.^[^ ^19^ ^]^ Under physiological conditions, ROS levels are kept stable by highly evolutionarily conserved antioxidant systems, primarily consisting of enzymes including the catalases (catalatic and peroxidatic), glutathione peroxidases, peroxiredoxins and the thioredoxin system.^[^ ^20^ ^]^ Should the production of oxidants exceed the capacity of these systems to remove them, a build‐up of ROS and oxidative stress will ensue.^[^ ^21^ ^]^ Susceptibility to oxidative stress is increased with age due to a reduction in the efficacy of oxidative stress systems, and this has been implicated in a number of age‐related neurological and cardiovascular diseases.^[^ ^22^ ^]^

The primary source of ROS (especially the superoxide anion radical) in cells are mitochondria,^[^ ^23^ ^]^ and dysfunctional mitochondria that are producing ATP inefficiently produce more mitochondrial ROS.^[^ ^24^ ^]^ Mitochondrial dysfunction therefore contributes greatly to oxidative stress and has been identified as a hallmark of ageing.^[^ ^19^ ^]^ Mice with impaired mitochondrial DNA (mtDNA) polymerase, resulting in increased mtDNA mutations, have a reduced lifespan and premature onset of ageing‐related phenotypes such as weight loss, hair loss, osteoporosis and reduced fertility^[^ ^25^ ^]^; this suggests that impaired mitochondrial function is sufficient to generate age‐related changes in mammals. Another major source of cellular ROS are NADPH oxidases, including NOX4, which has been implicated in another hallmark of ageing, namely senescence.^[^ ^26^, ^27^, ^28^ ^]^ NOX4 may represent a possible therapeutic target to reduce ROS production in age‐associated diseases such as cardiovascular disease,^[^ ^29^ ^]^ and to prevent the premature ageing phenotype seen in cancer survivors treated with radiotherapy.^[^ ^30^ ^]^ The association between ROS production by NOX4 and ageing phenotypes provides further evidence of the importance of oxidative stress in ageing.

Oxidative stress can result in the accumulation of oxidatively modified proteins, which can cause proteins to become conformationally unstable and prone to aggregation.^[^ ^31^ ^]^ This will increase the demand on the autophagy system as the formation of misfolded proteins requiring degradation increases; however, since autophagic activity decreases with age,^[^ ^5^ ^]^ the ability of the autophagy system to respond to any increase in misfolded proteins is reduced, resulting in the loss of proteostasis seen in ageing. Autophagy is also crucial in mitochondrial quality control, with the clearance of damaged mitochondria by autophagy (known as mitophagy) believed to play an important role in cellular homeostasis.^[^ ^32^ ^]^ It has been shown that impairment of autophagy resulting in reduced mitophagy activity causes mitochondrial dysfunction and excessive ROS production, triggering detrimental metabolic collapse in cells and inducing cell death.^[^ ^33^, ^34^ ^]^ This strongly suggests that mitophagy is the key machinery to regulate oxidative stress through mitochondrial quality control, by eliminating ROS‐releasing damaged mitochondria. Two proteins that are important in the targeting of mitochondria for mitophagy are the mitochondrially localised kinase PINK1 and the cytosolic E3 ubiquitin ligase Parkin. Following mitochondrial damage, PINK1 is activated on the surface of damaged mitochondria, where it phosphorylates ubiquitin and Parkin. Phosphorylated ubiquitin and Parkin bind, activating Parkin and leading to ubiquitin conjugation and ubiquitin chain formation on the surface of mitochondria.^[^ ^35^ ^]^ This allows dysfunctional mitochondria to be targeted for degradation.

Despite the importance of autophagy in oxidative stress, some autophagy proteins have been shown to be negatively regulated under oxidative stress conditions. Oxidation of the key autophagy‐related proteins ATG3 and ATG7 inhibits their function, and therefore suppresses autophagy.^[^ ^36^ ^]^ Mitophagy may be especially vulnerable, as oxidation impairs the function of PINK1 and Parkin.^[^ ^37^ ^]^ Inhibition of autophagy (especially mitophagy) by oxidative stress can therefore lead to a feedback loop, with increased oxidation of autophagy proteins impairing mitophagy. This would result in less efficient degradation of dysfunctional mitochondria with inefficient ATP production which would generate more ROS, increasing both protein misfolding, and therefore the workload of autophagy proteins, and the oxidation of these proteins, further impairing their function. This poses a particular challenge to longevous organisms, particularly humans.

In contrast to autophagy/mitophagy proteins that are downregulated by oxidation, some autophagy proteins can be activated by oxidation, such as ATG4^[^ ^38^, ^39^ ^]^ and the transcription factor TFEB, which is a crucial regulator of lysosome biogenesis^[^ ^40^ ^]^ and shows increased transcription and translocation to the nucleus under oxidative stress conditions.^[^ ^41^ ^]^ Emerging evidence also reveals that some selective autophagy receptors (SARs) can be activated by oxidation through their oxidation‐mediated oligomerisation, promoting the efficient activation of autophagy. In this review, we hypothesise that autophagy evolution has enabled humans to acquire defence mechanisms against oxidative stress; specifically, that SARs have evolved to upregulate autophagy in response to oxidative stress.

## HYPOTHESIS: EVOLUTION OF SELECTIVE AUTOPHAGY RECEPTORS ENABLES RESILIENCE AGAINST OXIDATIVE STRESS IN HUMANS

SARs recruit ubiquitinated cargoes for degradation by binding to ubiquitin with their ubiquitin binding domains (UBD) and the autophagosome component LC3 with their LC3‐interacting region (LIR) motifs.^[^ ^42^ ^]^ Whilst many SARs have been identified in various forms of selective autophagy,^[^ ^43^ ^]^ accumulating evidence suggests that certain SARs, in particular p62/SQSTM1 and NDP52 ***/*** CALCOCO2, are activated by oxidation, enabling them to increase autophagic activity in response to oxidative stress.^[^ ^44^, ^45^ ^]^ This response to oxidative stress appears to have been acquired relatively late in the evolution of these receptors and may have an important role in human longevity.

### Oxidation of p62 and autophagy with implications for ageing

**FIGURE 1:**
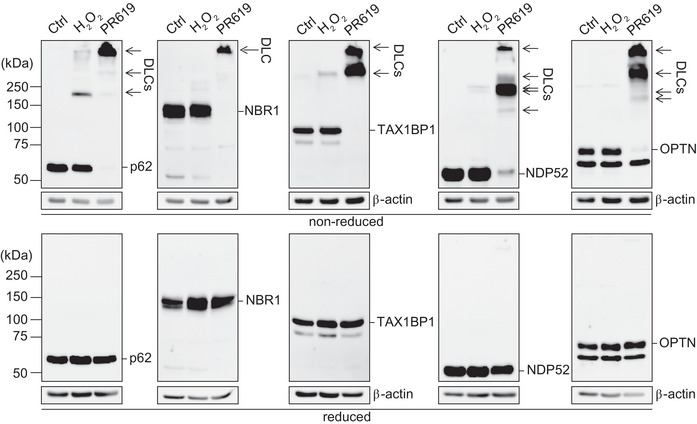
DLC formation of 5 SARs in response to oxidative stress induced by H_2_O_2_ or a redox cycler PR619.^[^ ^44^ ^]^ Immunoblotting of endogenous SARs in HeLa cells treated with H_2_O_2_ (5 mM, 1 min) or PR‐619 (10 μM, 10 min) in either non‐reducing or reducing (2.5% β‐mercaptoethanol) conditions. All SARs formed higher molecular bands (denoted as arrows) which were abolished in the reduced conditions, indicating the ability of the SARs to form DLCs in response to oxidative stress. Full methodology can be found in E.G. Otten (2017).^[^ ^70^ ^]^

**FIGURE 2:**
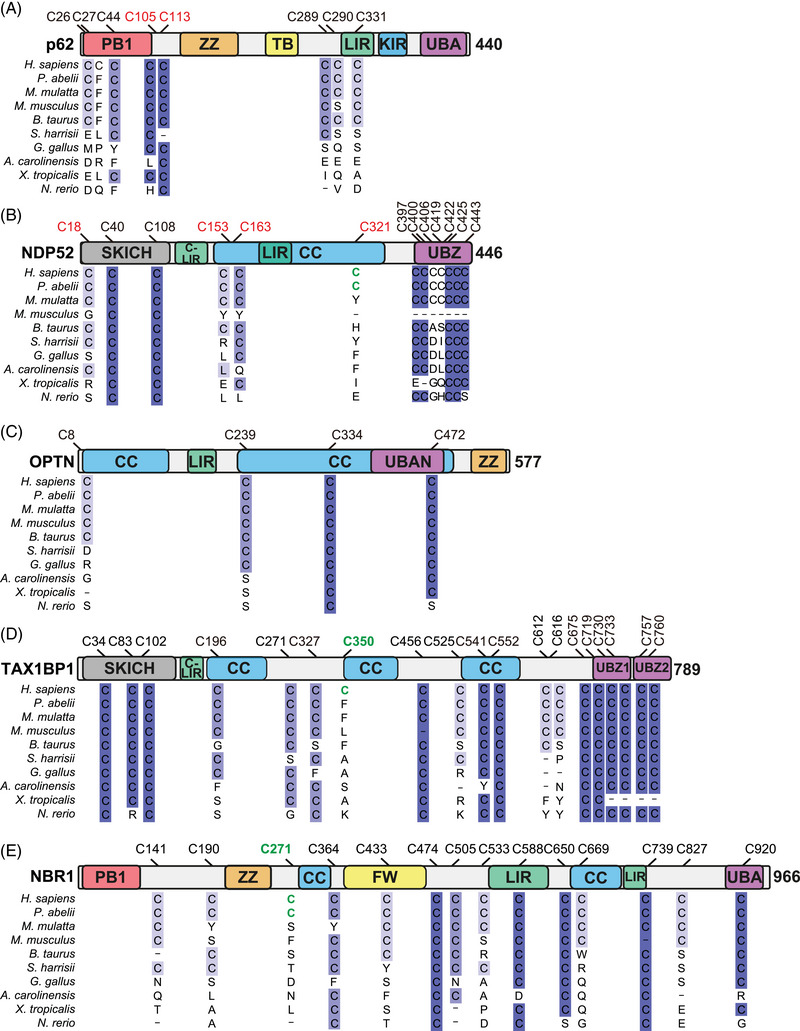
Schematic diagrams of the domain structure with multiple sequence alignment of OPTN, TAX1BP1 and NBR1. Schematic diagrams depicting the domain structure and location of cysteine residues with multiple sequence alignment of p62 (A), NDP52 (B), OPTN (C), TAX1BP1 (D) and NBR1 (E) across different species. Cysteine residues located in the ZZ domains, which are highly conserved across species, are not shown. Red indicates redox‐sensitive cysteine residues, and green indicates cysteine residues present only in human and orangutan. Protein sequences were obtained from NCBI and multiple alignment was carried out using Clustal Omega with default parameters. The resulting alignment was visualised using JalView (ver 2.11.2.6), and conservation is indicated by colour gradation from light to dark blue with conservation below 30% indicated in white. Abbreviation: CC, coiled‐coil; FW, four‐tryptophan; KIR, KEAP1‐interacting region LIR, LC3‐interacting region motif; PB1, Phox and Bem1; SKICH, SKIP carboxyl homology; TB, TRAF6‐binding domain; UBA, ubiquitin‐associated; UBAN, ubiquitin binding in ABIN and NEMO; UBZ, ubiquitin‐binding zinc finger; ZZ, ZZ‐type zinc finger.

p62 was the first SAR to be discovered, and remains the most widely studied.^[^ ^46^ ^]^ p62 self‐oligomerises by the binding of PB1 domains and can form a flexible filamentous helical scaffold that binds to LC3 and ubiquitinated protein cargoes, allowing it to mediate the autophagic degradation of protein aggregates (known as aggrephagy).^[^ ^47^ ^]^ Formation of p62 oligomers and aggregates promotes interaction with LC3^[^ ^48^ ^]^ and allows filaments to form clusters with ubiquitinated substrates, enabling them to channel these cargoes into autophagy.^[^ ^49^ ^]^ Oxidation at cysteine residues 105 and 113, which lie within a disordered region adjacent to the PB1 domain, promotes the formation of disulphide‐linked conjugates (DLCs) between p62 proteins, facilitating oligomerisation of p62 and enhancing autophagy (Figures 1 and 2A). This response is not present in invertebrates; the *Drosophila* p62 homologue Ref(2)P does not form DLCs in response to oxidation, and introducing oxidation‐sensitive cysteine residues into Ref(2)P increases autophagic flux and oxidative stress resistance in flies. Furthermore, the mutation of p62 identified as causative in the neurodegenerative disease amyotrophic lateral sclerosis (ALS), K102E, impairs p62 DLC formation and autophagy.^[^ ^44^ ^]^ These findings suggest that p62 has evolutionarily acquired the ability to respond to oxidative stress in vertebrates, and that impairment of this response is associated with age‐related neurodegeneration.^[^ ^50^ ^]^

Whilst p62 is not essential for PINK1/Parkin‐mediated damage‐induced mitophagy, recent evidence shows that p62 acts as a receptor for basal mitophagy.^[^ ^51^, ^52^ ^]^ p62 was shown to directly interact with the integral pore‐forming protein SAMM50 and to promote the degradation of the sorting and assembly machinery (SAM) and the mitochondrial contact site and cristae organising system (MICOS) components. The SAM is found in the outer mitochondrial membrane, and acts as an essential protein translocator, mediating the integration and assembly of β‐barrel proteins in the outer membrane,^[^ ^53^, ^54^ ^]^ whilst the MICOS is required for the formation of cristae junctions in the inner mitochondrial membrane.^[^ ^55^ ^]^ SAMM50 forms part of the SAM complex and interacts with the MICOS complex to sustain cristae organisation.^[^ ^56^ ^]^ p62 was shown to mediate basal piecemeal mitophagy via SAMM50, in which “worn out” MICOS and SAM complexes are replaced.

Another evidence for the link between basal mitophagy and p62 has been found in human primary dermal fibroblasts (HDFs) which display high levels of mitophagy at basal state.^[^ ^57^ ^]^ In HDFs, knockdown of p62 results in the reduction of basal mitophagy, whilst re‐introduction of p62 wild‐type but not oxidation insensitive p62 (C105A, 113A) restores mitophagy, indicating that p62 oxidation is involved in the activation of at least some types of mitophagy. Interestingly, the p62‐mediated basal mitophagy plays a key role in the prevention of cellular senescence, as suppression of mitophagy by genetic knockdown of PINK1, Parkin or p62 induces senescence and conversely, activation of p62‐dependent mitophagy using a small molecule rescues senescence phenotypes.^[^ ^57^ ^]^ These results suggest that in oxidative stress conditions, oxidation‐sensitive p62 contributes to mitochondrial quality control by eliminating portions of mitochondrial network that are potentially harmful to cells, thereby improving mitochondrial function and reducing mitochondrial ROS production.

As well as responding to oxidative stress by increasing autophagic flux, p62 can also affect the expression of antioxidant, cytoprotective and detoxifying enzymes by acting on the transcriptional regulator NRF2. NRF2 is crucial for cellular redox homeostasis due to its role in regulating the expression of antioxidant systems, as well as its importance for mitochondrial function,^[^ ^58^ ^]^ with NRF2‐deficient cells showing mitochondrial depolarisation and impaired respiration, along with increased oxidative stress.^[^ ^59^ ^]^ NRF2 activity is repressed by KEAP1, which forms the Cullin‐3‐type ubiquitin ligase complex that targets NRF2 for sequestration, ubiquitination and degradation,^[^ ^60^ ^]^ with inhibition of the KEAP1‐NRF2 interaction resulting in NRF2 activation.^[^ ^61^ ^]^ p62 contains a KEAP1‐binding region (KIR) that allows it to interact with the NRF2‐binding site on KEAP1, thereby inhibiting their interaction in a competitive manner and stabilising NRF2, resulting in transcriptional activation of NRF2 target genes.^[^ ^62^, ^63^ ^]^ There is also evidence that the target genes of NRF2 include p62, with NRF2 binding to an antioxidant response element (ARE) in the p62 promoter. This creates a positive feedback loop, in which p62 is activated under oxidative stress conditions via the oxidation of key cysteine residues and DLC formation; activated p62 then prevents KEAP‐1 dependent NRF2 degradation, increasing expression of NRF2 target genes including p62 itself.^[^ ^64^ ^]^ This will allow for continued formation of p62 oligomers to mediate autophagy (including mitophagy) and NRF2 stabilisation, even as p62 is degraded at a faster rate due to elevated autophagy in oxidative stress conditions. Notably, the positive feedback would not exist in yeast and flies since yeast lack p62, and Ref(2)P in files is redox‐insensitive.^[^ ^44^ ^]^ This may suggest vertebrates evolutionarily acquired resilience of the p62‐NRF2 axis to oxidative stress.

### Evolution of NDP52 promoting efficient activation of mitophagy

NDP52 is a SAR that has been shown to be particularly important in mitophagy, with PINK1 recruiting NDP52 to mitochondria independently of Parkin (along with another SAR, optineurin), where it interacts with the autophagy factors ULK1, DFCP1 and WIPI1.^[^ ^51^ ^]^ Re‐expression of NDP52, optineurin (OPTN), or, to a lesser extent, TAX1BP1, restored mitophagy in response to mitochondrial damage in cells in which five SARs (p62, NDP52, TAX1BP1, OPTN and NBR1) had been knocked out (pentaKO cells).^[^ ^51^ ^]^ This indicates that NDP52 and OPTN are the key mitophagy receptors, and thereby key regulators of the mitochondrial niche. Given that, as mentioned previously, mitochondria are the primary source of cellular ROS, with mitochondrial dysfunction leading to elevated ROS production, these mitophagy receptors are likely to be involved in the clearance of dysfunctional mitochondria to alleviate oxidative stress.

However, intriguingly, only NDP52 forms DLCs upon mitochondrial damage induced by mitochondrial depolarisation or accumulation of misfolded proteins in mitochondria, although OPTN can form DLCs in response to general oxidative stress conditions (such as exposure to hydrogen peroxide),^[^ ^45^ ^]^ suggesting a specific role for NDP52 in sensing ROS from mitochondria. This NDP52 DLC formation is mediated by cysteine residues (C18, C153, C163 and C321) that, similarly to those seen in p62, promote self‐oligomerisation of NDP52 upon oxidation (Figure 2B). Structurally, three of these residues, C153, 163 and 321, are located in the coiled coil (CC) domain that mediates protein dimerisation.^[^ ^65^ ^]^ Computational modelling indicated that C163 and C321 form C163‐C163 and C321‐C321 bonds within the dimer whilst C153 is predicted to be positioned away from the dimer interface and can be available for crosslinking between the dimers. In addition to C153, C18 within the SKIP carboxyl homology (SKICH) domain can also contribute to NDP52 oligomerisation. Indeed, mutations in these cysteine residues abrogate its ability to form tetramer and higher order oligomer species.^[^ ^45^ ^]^ Importantly, removing redox‐sensitivity of NDP52 by the mutations or pre‐treatment of mitochondrial ROS‐specific antioxidant, MitoQ, significant delays the onset of mitophagy after mitochondrial damage. At the same time, the NDP52 mutant fails to recruit LC3 to damaged mitochondria and its binding of the ULK1 autophagy initiator complex is reduced,^[^ ^66^ ^]^ indicating that oxidation‐sensitive NDP52 coordinates both autophagy initiation and autophagosome recruitment to the damaged mitochondria. Taken together, oxidation of NDP52 and subsequent oligomerisation may provide scaffold for autophagy/mitophagy machinery proteins, which are required for the rapid and efficient activation of mitophagy.

Notably, the redox‐sensitive cysteine residues were acquired late during human evolution, with all four residues present in apes and not found in shorter‐lived mammals such as mice. Introduction of human NDP52 to mouse cells is sufficient to initiate ROS‐dependent mitophagy.^[^ ^45^ ^]^ This raises the possibility that redox sensing mediated by NDP52 during mitophagy has evolved as a mechanism of oxidative stress response, which may be an adaptation and a contributor to the longer lifespan in primates including humans.

### Oxidation of other SARs and their possible functions

Several studies have shown that OPTN can self‐oligomerise,^[^ ^45^, ^67^, ^68^ ^]^ and that OPTN trimerises in response to oxidative stress.^[^ ^69^ ^]^ Indeed, we observed DLC formation in response to oxidative stress by OPTN as well as NBR1 and TAX1BP1, in addition to p62 and NDP52 (Figure 1).^[^ ^70^ ^]^ As mentioned earlier, OPTN re‐expression is sufficient to restore mitophagy in pentaKO cells, along with NDP52.^[^ ^51^ ^]^ However, mitochondrial ROS production induced by oligomycin and antimycin A treatment which is sufficient to trigger NDP52 DLC‐mediated PINK1/Parkin mitophagy is insufficient to induce OPTN DLC formation, suggesting that OPTN does not form DLCs as readily as NDP52.^[^ ^45^ ^]^ In contrast to NDP52, in which redox sensitive cysteine residues are only present in primates, including modern humans, two cysteine residues (C239 and C334) within the CC domain of OPTN are well conserved from bird to human (Figure 2C), which may reflect the differing sensitivity between NDP52 and OPTN against mitochondrial damage. Nevertheless, the effect of OPTN self‐oligomerisation on its activity, and the function of OPTN trimers formed in response to oxidative stress, remain unclear. As such, future studies to determine how OPTN oxidation and aggregation affects its role in autophagy, and especially mitophagy, may be useful to illuminate any possible role of OPTN in responding to oxidative stress.

**FIGURE 3:**
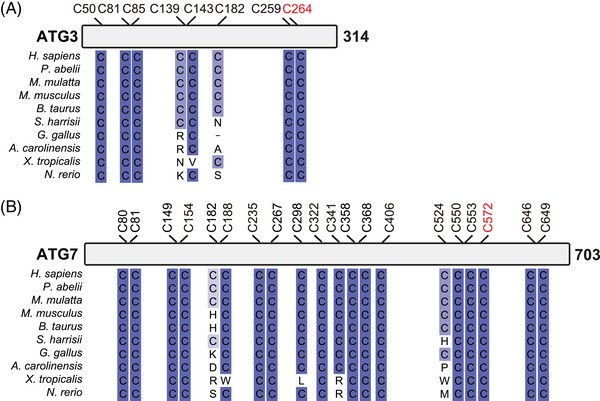
Schematic diagrams of multiple sequence alignment of ATG3 and ATG7. Schematic diagrams depicting the location of cysteine residues with multiple sequence alignment of ATG3 (A) and ATG7 (B) across different species. Red indicates redox‐sensitive cysteine residues.^[^ ^36^ ^]^ Protein sequences were obtained from NCBI and multiple alignment was carried out using Clustal Omega with default parameters. The resulting alignment was visualised using JalView (ver 2.11.2.6), and conservation is indicated by colour gradation from light to dark blue with conservation below 30% indicated in white.

TAX1BP1 can direct aggrephagy of ubiquitinated protein conjugates, with lack of TAX1BP1 in mice leading to protein aggregate accumulation in the brain^[^ ^71^ ^]^; whether this is dependent on oxidation‐induced DLC formation is unclear. Interestingly, alignment shows that C350 within the second of the three CC domains of TAX1BP1 exists only in humans, whilst three cysteine residues (C196, C541 and C552) are relatively conserved (Figure 2D). Investigating whether there are differences between human and other species’ TAX1BP1 in their functions in selective autophagy and redox sensitivity in response to oxidative stress may reveal a factor contributing to human longevity. Similarly, C271 of NBR1, located near the first CC domain, is present in human and orangutan but not in macaque and lower species (Figure 2E). There is currently no evidence that oxidation of NBR1 induces selective autophagy in response to oxidative stress, which may also be an interesting research avenue. Importantly, the evolutional diversity of cysteine residues of SARs is particularly apparent when compared to core autophagy proteins ATG3 and ATG7.^[^ ^36^ ^]^ Indeed, redox‐sensitive Cys264 of ATG3 and Cys572 of ATG7 are perfectly conserved across species, which may reinforce our hypothesis of evolution of SARs in response to oxidative stress (Figure 3A,B).

It remains unknown whether oligomerisation of NBR1 and TAX1BP1 functions as a redox sensor in a specific type of autophagy, as well as their evolutional diversity or conservation of cysteine residues. However, these SARs have been shown to interact with p62, with NBR1 promoting ubiquitin condensate formation by p62 and recruiting TAX1BP1, which in turn recruits the autophagosome scaffold protein FIP200, promoting p62‐ubiquitin condensate degradation.^[^ ^72^ ^]^ This may explain why, even though p62 is not sufficient to re‐establish mitophagy in pentaKO cells, as described earlier,^[^ ^51^ ^]^ removal of p62 can reduce mitophagy^[^ ^52^, ^73^ ^]^; this suggests that p62 is necessary for some forms of mitophagy, but could dependent on other SARs, most likely NBR1 and TAX1BP1. As NDP52 and OPTN are sufficient to carry out mitophagy in response to mitochondrial damage by oligomycin and antimycin A treatment,^[^ ^51^ ^]^ whereas p62 is believed to mediate basal “piecemeal” mitophagy,^[^ ^52^ ^]^ it is possible that these SARs act via different mitophagy pathways to mediate different types of mitophagy. Variants of mitophagy have already been proposed, with damage‐induced (or Type 2) mitophagy distinguished from mitochondria‐derived vesicle formation (micromitophagy or Type 3 mitophagy).^[^ ^74^ ^]^ NDP52 and OPTN appear to be responsible for damage‐induced mitophagy, whereas micromitophagy may be dependent on p62, NBR1 and TAX1BP1, with p62 targeting dysfunctional mitochondrial components (such as the SAM and MICOS complexes) for vesicle formation. The involvement of 3 different SARs may allow for tighter regulation of this process, rather than the less regulated response to high levels of oxidative damage that Type 2 mitophagy represents. Whilst oxidation of cysteine residues in p62 has been shown to promote its self‐oligomerisation, whether it affects the interaction between p62 and other SARs is still unknown; determining this may help to further elucidate how p62 responds to oxidative stress.

**FIGURE 4:**
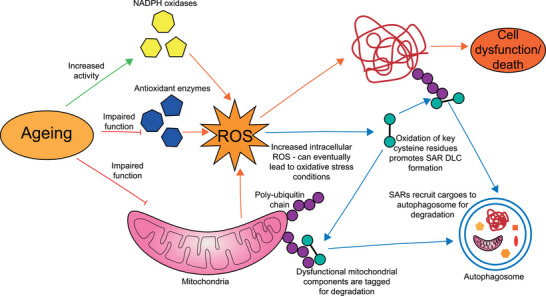
Proposed interplay between selective autophagy and age‐related oxidative stress. Ageing results in the impaired function of mitochondria and antioxidant systems, and increases NOX activity (in particular NOX4). This results in elevated intracellular ROS, which can lead to misfolding and aggregation of oxidated proteins, resulting in cellular dysfunction and eventually death. However, SARs can form DLCs following oxidation of specific cysteine residues, allowing them to recruit protein aggregates to the autophagosome for degradation by aggrephagy. SAR DLCs can also improve mitochondrial function by mediating mitophagy, thereby reducing mitochondrial ROS production and restoring redox balance. The exact SARs involved may vary depending on the form of autophagy taking place, for example, p62 and TAX1BP1 are heavily implicated in aggrephagy,^[^ ^49^, ^71^ ^]^ whilst NDP52 and OPTN are responsible for damage‐induced mitophagy.^[^ ^51^ ^]^

In addition, activation of selective autophagy via oxidation of SARs may be regulated in tissue‐specific manner due to their differing abundance. For instance, OPTN and TAX1BP1 are abundantly expressed in brain where there is little to no NDP52.^[^ ^51^, ^71^ ^]^ This raises the possibility that OPTN and/or TAX1BP1 sense ROS and initiates mitophagy in the brain, similar to the role of NDP52 found in HeLa cells.^[^ ^45^ ^]^ Thus, future investigations integrating individual functions of SARs in specific type of selective autophagy in different cell types and tissues will help to fully delineate how selective autophagy combats oxidative stress and contribute to cellular homeostasis. An overview of how oxidation and oligomerisation of SARs maintain cellular homeostasis by inducing autophagic degradation of mitochondria and protein aggregates is provided in Figure 4.

### Significance in age‐related neurodegenerative diseases

The brain, the function of which is essential for human life, is especially susceptible to oxidative stress due to its high energy demand, consuming around 20% of oxygen supplied by the respiratory system despite making up around 2% of body weight. In addition, neuronal cell membranes are rich in unsaturated fatty acids that are prone to oxidation, and antioxidant defences such as catalases are expressed at low levels.^[^ ^75^ ^]^ Considering that autophagy has an essential function for the elimination of ROS‐releasing mitochondria and aggregate‐prone proteins which are potentially harmful to neuronal cells, the ability of SARs to upregulate autophagy in response to oxidative stress likely contributes to human longevity. This may also provide a potential therapeutic target in age‐related disorders associated with ageing, such as Alzheimer's disease (AD) and Parkinson's disease (PD). These proteinopathies are characterised by the build‐up of misfolded protein aggregates and mitochondrial damage, resulting in neuronal cell death, and both oxidative stress and impaired autophagy are believed to play an important role in the progression of these diseases.^[^ ^76^, ^77^ ^]^

In proteinopathies, oxidative stress can induce the formation of pathogenic forms of a protein, which can have a cytotoxic effect and further increase ROS overproduction.^[^ ^78^ ^]^ p62 may be of particular interest as a target for preventing neurodegeneration by upregulating ROS‐induced autophagy, as p62 knockout mice show hyperphosphorylation and aggregation of tau, which is a key hallmark of AD. These mice also show neurodegeneration and cognitive decline.^[^ ^79^ ^]^ However, the aggregates formed were not the paired helical filaments seen in AD, instead representing a pre‐tangle form of tau. This suggests that p62 is necessary for aggregate formation as well as misfolded protein degradation, which is further supported by evidence that Ref(2)P is required for protein aggregate formation in *Drosophila* brain.^[^ ^80^ ^]^ This role of p62 may also be neuroprotective, as pre‐fibrillar tau oligomers are thought to represent the toxic species in AD, rather than fibrillar tau.^[^ ^81^ ^]^

The ability of p62 to respond to oxidative stress and protein misfolding may be impaired in neurodegenerative diseases due to reduced p62 transcription, as oxidative DNA damage of the p62 promoter region is common in brain samples from neurodegenerative disease patients. This results in reduced p62 expression, leading to the further disruption of redox homeostasis through the inactivation of NRF2.^[^ ^82^ ^]^ Mutations in p62 are associated with neurodegenerative diseases ALS and frontotemporal lobar degeneration (FTLD),^[^ ^44^ ^]^ including in the KIR region.^[^ ^83^ ^]^ These disease‐linked mutations disturb selective autophagy and NRF2‐mediated anti‐oxidative stress responses,^[^ ^84^ ^]^ further supporting the link between disruption of NRF2 activation via p62 and neurodegeneration. This demonstrates that impairment of the p62 response to oxidative stress reduces neuronal cell survival, consistent with the hypothesis that the oxidative stress response by p62 supports human longevity. Considering the activation of NRF2 via inhibition of the KEAP1‐NRF2 interaction has been found to be neuroprotective in a cellular PD model,^[^ ^61^ ^]^ the regulation of cellular redox homeostasis and mitochondrial function by NRF2 presents a possible therapeutic target in PD and other neurodegenerative diseases associated with p62 deregulation.

Mitochondrial dysfunction resulting in elevated ROS production may be a key player in the pathogenesis of disorders such as PD and AD, in which markers of oxidative stress are evident.^[^ ^85^, ^86^ ^]^ The “mitochondrial cascade hypothesis” proposes that late‐onset, sporadic AD results from ROS production by damaged mitochondria,^[^ ^87^ ^]^ and stimulation of mitophagy can reduce β‐amyloid and tau pathology in human neuronal cells and animal AD models, and reverse cognitive deficits in these models.^[^ ^88^ ^]^ Impaired mitophagy can also lead to PD development; mutations of both Parkin^[^ ^89^ ^]^ and PINK1^[^ ^90^ ^]^ can result in early‐onset, hereditary PD, whilst post‐mortem samples of substantia nigra from PD patients show impaired mitochondrial Complex I,^[^ ^91^ ^]^ suggesting that dysfunctional mitochondria are not being cleared. This will result in increased mitochondrial ROS production in PD, which can lead to neurodegeneration, such as by increasing assembly of the neuronal NLRP3 inflammasome.^[^ ^92^ ^]^ Thus, promoting mitophagy by inducing oligomerisation of p62 and potentially other SARs using small molecules, which are being developed for p62‐mediated autophagy,^[^ ^48^ ^]^ could be a new strategy to treat these diseases which manifest mitochondrial dysfunction.^[^ ^57^ ^]^

Another age‐associated disorder in which activation of SARs by oxidative stress may present a novel therapeutic target is glaucoma, a major cause of blindness resulting from optic nerve damage. Oxidative stress is an important risk factor in the pathogenesis of primary open‐angle glaucoma (POAG), with oxidative stress markers elevated in POAG patients.^[^ ^93^ ^]^ Similarly, oxidative DNA damage after hydrogen peroxide treatment is greater in POAG patients than in healthy controls, and antioxidant enzyme levels lower.^[^ ^94^ ^]^ The E50K OPTN mutation causes familial POAG and disables OPTN oligomerisation.^[^ ^95^ ^]^ This, combined with the evidence of oxidative stress in POAG, suggests that the self‐oligomerisation response of OPTN to oxidative stress may be important in preventing POAG, and could represent a promising therapeutic target.

## CONCLUSION AND FUTURE DIRECTIONS

The accumulation of oxidative stress in aged organisms has long been seen as a key contributor to ageing and is thought to play a critical role in a variety of age‐associated diseases. Oxidation impairs the function of several evolutionarily conserved autophagy proteins, reducing the capacity of the autophagy system to degrade both the dysfunctional mitochondria that produce most cellular ROS and the misfolded and damaged proteins that result from excessive ROS production. It therefore follows that long lived organisms, including humans, may have evolved mechanisms to increase autophagic function in response to oxidative stress. We propose that in humans these mechanisms involve activation of SARs by oxidation of evolutionarily acquired cysteine residues, which promotes self‐oligomerisation. Both p62 and NDP52 respond to oxidation of key cysteine residues with self‐oligomerisation and activation. Intriguingly, introduction of these residues into Ref(2)P increases fly survival in stress conditions, and introduction of human NDP52 is sufficient to initiate redox‐responsive mitophagy in mouse cells.^[^ ^44^, ^45^ ^]^ These reverse engineering approaches are starting to highlight the importance of selective autophagy as a mechanism of oxidative stress response for human physiology and longevity.

Several experimental strategies for future research could provide further support for this hypothesis; this includes analysis of protein oxidation by DLC detection when examining autophagy activation in response to oxidative stress. Further establishing the link between oxidation of SARs, DLC formation and autophagy activation would provide critical experimental evidence for this hypothesis. Additionally, testing the proposed pathway across cell lines from different species, such as humans and mice, would improve our understanding of the evolution of this response and at what stage SARs acquired the ability to respond to oxidative stress.

Many unanswered questions remain regarding the role of SARs in responding to oxidative stress and in ageing that future research should aim to address. One example outlined in this article is the potential involvement of OPTN, NBR1 and TAX1BP1 as redox sensors in specific types of selective autophagy. Indeed, the functions of each SAR, such as the extent to which each can control different forms of autophagy, including various types of mitophagy, are still not fully understood. Finally, whether SARs could be targeted in age‐associated diseases that are associated with oxidative stress and impaired autophagy, which includes numerous neurodegenerative diseases, is also an area where further research is required.

## AUTHOR CONTRIBUTIONS

J.R., T.K. and V.I.K. wrote the original draft. J.R., T.K., E.G.O. and V.I.K. reviewed and edited the manuscript. E.G.O. generated immunoblotting data. T.K. performed multiple sequence alignment. J.R. generated diagram illustration. T.K. and V.I.K. acquired funding.

## CONFLICT OF INTEREST STATEMENT

V.I.K. is a Scientific Advisor for Longaevus Technologies.

## Data Availability

Any information reported in this paper is available from the corresponding author (V.I.K.) upon request.
